# Association of Number of Comorbid Conditions and Pain among United States Adults

**DOI:** 10.3390/diseases12070147

**Published:** 2024-07-10

**Authors:** David R. Axon, Becka Eckert

**Affiliations:** Department of Pharmacy Practice and Science, R. Ken Coit College of Pharmacy, The University of Arizona, 1295 North Martin Avenue, P.O. Box 210202, Tucson, AZ 85721, USA; beckert@arizona.edu

**Keywords:** multimorbidity, comorbid conditions, pain, United States adults

## Abstract

Studies have explored the association of particular conditions, or combinations of conditions, and pain among specific populations. However, there is limited information regarding the association of the number of comorbid conditions, as well as other demographic, economic, health, and limitation variables, with pain among adults in the United States. This cross-sectional database study aimed to examine the relationships between number of comorbid conditions (including cancer, arthritis, joint pain, stroke, heart attack, angina, coronary heart disease, high cholesterol, high blood pressure, other heart diseases, diabetes, asthma, chronic bronchitis, and emphysema), demographic, economic, health, and limitation variables with pain among United States adults using 2021 Medical Expenditure Panel Survey data. A multivariable logistic model assessed the association between the number of comorbid conditions (≥6, 5, 4, 3, 2, 1, versus 0 conditions) and quite a bit/extreme (versus little/moderate) pain, adjusting for demographic, economic, health, and limitation variables. The study found that greater numbers of comorbid conditions were associated with higher odds of quite a bit or extreme pain. In addition, age, education, employment, income, overall health, regular physical activity, and three limitation variables were each associated with pain in the multivariable model. These findings offer insight into the association between number of comorbid conditions and other variables with pain and suggest areas where interventions may be helpful to help improve pain outcomes for United States adults.

## 1. Introduction

Pain is a complex process that has negative consequences to individual persons, their families, and society as a whole [1]. Pain lasting three months or longer affects the activities of daily living, ability to work, and quality of life for millions of United States (US) residents [2]. Pain is defined by the International Association for the Study of Pain as “an unpleasant sensory and emotional experience associated with, or resembling that associated with, actual or potential tissue damage” [3]. Pain may be described and assessed in different ways [4] and can be acute or chronic [5]. Acute pain typically has a sudden onset, short duration, and is provoked by a cause [4]. Chronic pain can be considered a disease itself when it is recurrent or lasts longer than three months [4]. The Verbal Rating Scale is a widely accepted method to describe pain according to severity (e.g., mild, moderate, or severe), and may also reflect patient perceptions and beliefs about their pain and its interference with their functioning [6].

Acute pain, such pain from an injury, can have short-term negative impacts on one’s mood, mobility, or functionality. Chronic pain can be detrimental to long-term physical and mental wellbeing [7]. Prolonged pain negatively affects sleep, brain function, and impairs cognitive processes [7]. Cardiovascular health and sexual health are also impacted by unresolved pain [7]. A survey performed throughout 17 countries, including the US, found a strong association between anxiety and mood disorders among people who reported pain at one or more sites. The prevalence of anxiety and mood disorders was positively correlated to the number of reported pain sites, with the lowest rates of disorders in people with no pain sites and the greatest reported rates of disorders in those with more than one pain site [8]. A study of older adults with arthritis found that lower back pain increases the risk of insomnia and psychological distress and is associated with mobility limitations and poorer overall health [9]. A qualitative study described comorbid conditions and health perception and found that diseases are experienced differently depending on the presence or absence of pain. One patient reported experiencing unresolved back pain that disrupted sleep, caused stress, and exacerbated depression, eventually leading to a cardiac event [10].

Chronic pain has a high disease burden among the US adult population [11]. The 2021 National Health Interview Survey found that over twenty percent of US adults reported experiencing chronic pain [2]. Respondents with chronic pain reported limitations in social and daily activities, and missed more workdays compared to those without chronic pain [7]. The impact of pain on health through multimorbidity and disability is estimated to cost the US USD 560–635 billion annually, surpassing the cost of care for heart disease, cancer, and diabetes combined [12]. It is estimated that pain causes about four billion workdays to be lost every year, at substantial cost to US productivity [13].

Comorbid conditions, including mood disorders (e.g., depression or anxiety), diabetes, and heart disease, are commonly associated with pain [14]. Datasets such as the Medical Expenditure Panel Survey (MEPS) used in the current study include several conditions of interest that are worthy of investigation among people with pain, including cancer, arthritis, joint pain, stroke, heart attack, angina, coronary heart disease, high cholesterol, high blood pressure, other heart diseases, diabetes, asthma, chronic bronchitis, and emphysema [15,16]. A study of 573 patients diagnosed with depression found over two-thirds of the patients reported having pain. Of those who experienced both depression and pain, patients who described their pain as severe had worse treatment outcomes than those with mild pain. This finding suggests that comorbid conditions and pain may be correlated with poor treatment outcomes [17]. An observational study of 993 people with diabetes found that patients who reported having pain had poorer management of their diabetes than those without pain. They also had a more difficult time following an exercise regimen and nutrition plan that could help improve diabetes outcomes [18]. Chronic pain is also a significant barrier for performing self-care related to disease management [18].

It has been documented that pain increases the likelihood of cardiovascular diseases [19]. A study of nearly a half a million patients found that pain as a risk factor for heart disease is comparable with that of diabetes [20]. Experiencing pain activates the sympathetic nervous system, releasing hormones that result in cardiovascular system stress. This added stress may cause an increase in blood pressure, chest pain, and cardiovascular complications [21].

Furthermore, several different demographics such as age, sex, education, income, and employment have been associated with pain [22]. One study found women are more likely to report pain compared to men and older adults have a higher likelihood of reporting pain than young adults [23]. People who are unemployed and those with an income of less than 200% of the federal poverty level were associated with reporting increased pain intensity [22].

Some studies have explored the association of a particular condition, or combination of conditions, with pain among specific populations [14,24,25,26,27]. However, there is limited information regarding the association between the number of comorbid conditions and other demographic factors with pain among adults in the US. Given this gap, there is a need to explore the relationship between comorbid conditions and pain among a demographically representative sample of US adults. Understanding these associations better could potentially lead to interventions that improve pain management strategies, reduce multimorbidity, and enhance quality of life. Therefore, this paper aims to examine the relationships between the number of comorbid conditions (including cancer, arthritis, joint pain, stroke, heart attack, angina, coronary heart disease, high cholesterol, high blood pressure, other heart diseases, diabetes, asthma, chronic bronchitis, and emphysema), demographic, economic, health, and limitation variables with pain among US adults.

## 2. Methods

This cross-sectional database study involved 2021 MEPS data, which collected demographic, economic, health, and limitation variables data for 28,336 participants. MEPS utilizes a panel design that involves interviewing a subsample of individuals from the National Health Interview Survey in five rounds over a period of two calendar years. The MEPS sampling framework enables the data to be representative of the national, non-institutionalized US population when weighted appropriately in analysis. MEPS is approved by an Institutional Review Board annually and all participants are required to provide informed consent before participating [28].

The study included the following eligible individuals: alive throughout 2021, aged 18 years or older, and had some degree of pain. The dependent variable was pain, which had the following five levels: extreme pain, quite a bit of pain, moderate pain, little pain, none. This was determined based on responses to the following question: during the past four weeks, how much did pain interfere with your normal work (including both work outside the home and housework)? [15,16]. Pain was classified as quite a bit/extreme or little/moderate in this analysis.

The independent variable was the number of comorbid conditions. The list of conditions included cancer, arthritis, joint pain, stroke, heart attack, angina, coronary heart disease, high cholesterol, high blood pressure, other heart diseases, diabetes, asthma, chronic bronchitis, and emphysema. The number of conditions per person was summed and then dichotomized to form the dependent variable [15,16]. The number of comorbid conditions for this study could range from 0 to ≥6.

**Figure 1:**
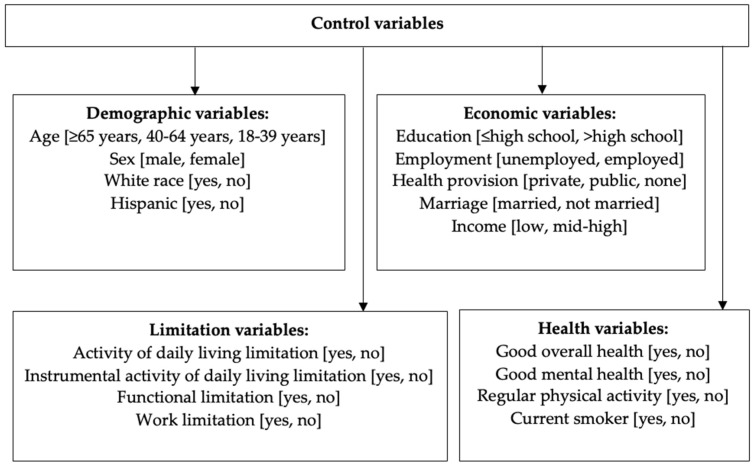
Framework of variables included in the adjusted analysis.

Control variables were included based on their availability in the dataset and their relevance to pain and comorbid conditions. Control variables consisted of demographic, economic, health, and limitation variables. Demographic variables included age (≥65 years, 40–64 years, 18–39 years), sex (male, female), White race (yes, no), and Hispanic (yes, no). Economic variables included education (≤high school, >high school), employment (unemployed, employed), health provision (private, public, none), marriage (married, not married), and income (low, mid-high). Health variables included good overall health (yes, no), good mental health (yes, no), regular physical activity (yes, no), and current smoker (yes, no). Limitation variables included activity of daily living limitation (ADL; yes, no), instrumental ADL limitation (yes, no), functional limitation (yes, no), and work limitation (yes, no) [15,16] (Figure 1).

Data analysis was conducted using SAS and utilized the SAS Survey Procedures (SAS Institute Inc., Cary, NC, USA). The characteristics of individuals with quite a bit/extreme pain versus little/moderate pain were compared using chi-squared tests. The proportion of people who had each condition was summarized and reported as a weighted percentage and confidence interval. An unadjusted logistic regression model assessed the association between the number of comorbid conditions and pain. Then, a multivariable logistic model assessed the association between number of comorbid conditions and pain, adjusting for the control variables. In both the unadjusted and adjusted models, quite a bit/extreme pain was modeled while little/moderate pain served as the reference group. The SAS SURVEYLOGISTIC procedure was used in the logistic regression models. An alpha value of 0.05 was selected a priori. Variables provided in the Medical Expenditure Panel Survey data file were utilized to maintain the cluster and strata of the data, as well as to obtain weighted population-based estimates. The University of Arizona Institutional Review Board approved this study (Study #00002451, 2 February 2023).

## 3. Results

**Figure 2:**
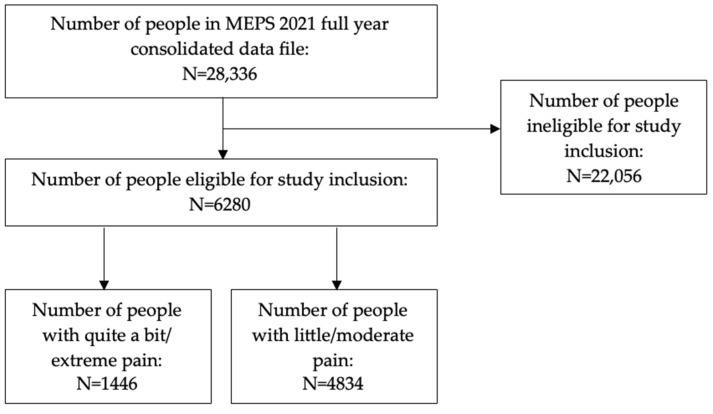
Subject eligibility diagram.

In all, the study included 6280 subjects, which represented a sample (weighted) of 89,314,769 US adults. Of these, 1446 had quite a bit/extreme pain, which represented a sample (weighted) of 17,306,210 (19.4%; 95% confidence interval [CI]: 18.0%, 20.7%), while 4834 had little/moderate pain, which represented a sample (weighted) of 72,008,559 (80.6%; 95% CI: 79.3%, 82.0%) (Figure 2).

Table 1 describes the characteristics of US adults in the study organized by quite a bit/extreme versus little/moderate pain status. There was a spread of number of comorbid conditions among the study sample. The most common number of comorbid conditions was 1 (20.4%), followed by 2 (19.6%), 3 (16.4%), 0 (14.0%), 4 (12.8%), ≥6 (8.9%), and 5 (7.9%). Overall, the most frequently reported age group was 40–64-year-olds (44.1%). Study participants were most often female (55.1%), White (79.4%), non-Hispanic (88.2%), had completed greater than high school education (57.1%), were employed (53.2%), had private health provision (60.1%), were married (53.2%), had mid-high income (69.4%), had good overall health (75.1%), had good mental health (83.0%), did not do regular physical exercise (54.4%), were non-smokers (85.6%), and had no ADL limitations (96.4%), no instrumental ADL limitations (93.4%), no functional limitations (72.5%), and no work limitations (80.5%). There were differences between groups for all variable except race and Hispanic status.

Table 2 delineates the weighted percentage of people with each comorbid condition. The most common conditions were hypertension, arthritis, hypercholesterolemia, and joint pain.

Table 3 displays unadjusted associations for number of chronic conditions and pain levels among US adults. Relative to adults with no comorbid conditions, greater numbers of comorbid conditions were associated with higher odds of quite a bit or extreme pain. Table 3 also presents adjusted associations for number of chronic conditions and pain levels among US adults. As with the unadjusted model, greater numbers of comorbid conditions were associated with higher odds of quite a bit or extreme pain in the adjusted model. In addition, age, education, employment, income, good overall health, regular physical activity, and three limitation variables (ADL, functional, and work) were each associated with higher odds of quite a bit or extreme pain.

## 4. Discussion

In this cross-sectional database study, we found that having greater numbers of comorbid conditions was associated with greater odds of experiencing quite a bit or extreme pain. Relative to having no comorbid conditions, each additional comorbid condition was associated with greater odds of experiencing quite a bit or extreme pain, with six or more comorbid conditions having the greatest odds of association with quite a bit or extreme pain. These findings are logical, as we would expect those with multiple comorbid conditions to have a greater likelihood of pain, either as a result of these comorbid conditions or due to poorer health. It has been discussed previously that US adults over 50 years old with quite a bit or extreme pain were associated with greater odds of multimorbidity, defined as ≥5 conditions, than US adults over 50 years old with little or moderate pain [29]. Our study findings indicate that an association exists between higher numbers of comorbid conditions and greater odds of reporting quite a bit or extreme pain across all US adults regardless of age, not just older US adults. This association is apparent in other contemporary studies as well. For instance, a survey among US Department of Veterans Affairs primary care patients found that those who reported having chronic pain had a greater number of health conditions [30]. Outside the US, a recent study of Canadians found that adults who had multiple co-occurring diseases were more likely to report pain, with each additional disease associated with an eight percent increase in the odds of reporting pain [31]. Another study using the Spanish National Health Survey data found that those with chronic obstructive pulmonary disease (COPD) were more likely to have chronic back pain than non-COPD controls after adjusting for relevant clinical variables including age and sex [32]. Our findings therefore add additional support for the association between multimorbidity and pain.

The findings of the current study have implications beyond pain for our understanding of morbidity and mortality among US adults. Comorbid diseases such as those included in the current study are known causes of morbidity and mortality among US adults [33]. Further work is warranted to prevent chronic diseases, modify poor lifestyle choices, and address social determinants of health that influence health outcomes [34]. Additional work may be needed to help better determine risk factors for morbidity and mortality among US adults. For instance, previous research has identified that a cumulative deficit index, which incorporated some comorbid conditions, among other things, was better able to identify and discriminate longevity and risk of death among vulnerable older US adults relative to phenotypic frailty and physiologic indices [35,36].

Our study also found associations between several demographic, economic, health, and limitation variables. Among the demographic variables, older age, specifically those 65 years and older, was associated with lower odds of experiencing quite a bit or extreme pain compared to those who were 18–39 years old. This may not seem like a logical finding, as older adults typically experience more pain due to age-related degenerative changes and multimorbidity [37]. One possible hypothesis for this finding is that older adults may have accepted their pain conditions and have adapted to their health state, therefore underreporting the interference that pain has on their lives. It has been previously discussed that older adults may underreport their pain if they believe pain is a normal component of ageing [37], which may be the case in our study. There was no statistically significant association with middle-aged adults versus younger-aged adults and the odds of experiencing pain. We found no association between sex, race (specifically White), or ethnicity (specifically Hispanic) and experiencing quite a bit or extreme pain. Some evidence suggests pain is more prevalent among women and that they are less likely to recover from chronic pain than men [38]. Recent studies have found nuanced differences in pain sensitivity and sex, with women having slightly lower pain thresholds and tolerances than men [38,39]. There is considerable evidence that Black individuals and other minority group members experience a greater burden of pain due to increased pain sensitivity and reduced pain tolerance compared to non-Hispanic Whites [40,41,42]. These differences were not represented in our findings. There are numerous psychosocial and biological mechanisms that contribute to differences in pain, and more research is needed in this area [39]. It was encouraging that there was no association with these demographic variables and pain, given that they are not modifiable.

Among the economic variables in this study, we found that having less than a high school education, unemployment and low income were each correlated to higher odds of experiencing quite a bit or extreme pain. These three variables (education, employment, and income) are typically related to one another, as those with higher education may have higher-paying jobs. Individuals of lower socioeconomic status are often disproportionately impacted by pain [43]. Those with lower income may struggle with aspects of healthy living, such as acquiring healthy food and prioritizing physical activity, and often have poorer health outcomes due to lack of healthcare resources [43]. Lower-paying jobs may be more physically demanding or have greater occupational hazards, predisposing an individual to the development of pain. Lower-income people with pain often have limited access to pain treatments to modify their living or work environments, thus experience more pain, and may be unable to improve their function [43]. Lower educational achievement has been associated with a higher prevalence of pain and more severe pain compared to those with more education [43]. A recent study investigating pain among patients with sickle cell disease found that those who were unemployed reported more frequent pain episodes and rated their pain as higher than those who were employed [44]. Research has historically shown that there is an association between unemployment and poorer health status, though this is not always demonstrated longitudinally [45]. There were no statistically significant associations between health insurance provision, either public or private, and experiencing pain compared to those without insurance. This is an encouraging finding as it suggests that there is no difference in pain based on the availability of health provision. This may also indicate that health insurance provides no significant benefit for patients who experience pain. A scathing discussion of America’s health insurance industry outlined the numerous ways insurers perpetuate pain through failing to reimburse providers, denying services and medications, and delaying treatment [46]. Chronic pain often lacks a clear etiology and has been viewed by health insurers as illegitimate, making it more difficult to access and pay for services related to treatment [46]. A recent study of US adults with pain found that a high-deductible health plan is associated with a reduced probability of receiving chronic pain treatment [47]. High-deductible health plans may inhibit access to first-line, nonpharmacologic treatment modalities [47]. In our study, we did not find any association between marital status and experiencing quite a bit or extreme pain. In 2018, Bjarnnes et al. suggested that the social support that marriage provides may improve pain and functioning; however, they did not find any association between marital status and pain perception among their post-operative patients [48]. Given this existing knowledge, the association of education, employment, and income with pain is unsurprising yet raises awareness of this issue for future consideration. Public health policies to help address these socio-economic differences may be warranted.

In this study, two health-related variables (good overall health and regular physical activity) were correlated with lower odds of experiencing quite a bit or extreme pain. This is a logical finding as people with pain typically have worse health status [38]. A recent study investigated the health status of patients with chronic low back pain and found that health-related quality of life was worse across several domains (physical function, general health, vitality) compared to those without pain [49]. Another study using the 2020 MEPS dataset also found that patients with quite a bit or extreme pain were associated with lower odds of reporting good health when compared to those with little pain [50]. The association between physical activity and pain is supported in the literature. Pain has previously been reported as a barrier to physical activity, but engaging in physical activity can improve symptoms and can be one of the best methods for managing pain [51]. Exercise reduces pain perception, improves mood, and reduces stress and depression, which are often associated with pain [51]. A recent study found that people with type 2 diabetes who experience severe pain were associated with decreased likelihood of being physically active [52]. This is an unfortunate finding, as physical activity has numerous benefits including improved glycemic control, cardiorespiratory fitness, and muscle strength [52]. Some patients, particularly those with fibromyalgia, experience heightened pain while engaging in exercise programs. This can lead to a sedentary lifestyle that worsens the painful conditions and makes treatment even more difficult [51]. Given that health status and exercise status are potentially modifiable factors for pain, particular attention should be applied to help people with pain improve their health and engage in regular physical activities, to the best of their ability depending on the nature of their health conditions. Specific physical activities may be recommended for people with specific health conditions so as to not aggravate the condition and worsen pain. For example, low-impact exercise, whether land-based or water-based, has been shown to decrease pain and improve functioning among people with osteoarthritis of weight-bearing joints [53]. Another study found that adults with fibromyalgia experienced more pain relief when utilizing water-based exercise compared to land-based or no physical exercise [54]. Although there was no association between the remaining health-related variables in this study (mental health status and smoking status) with pain, interventions or monitoring of these factors may still be recommended. Previous studies have suggested that smoking is a risk factor for chronic pain but that nicotine may have analgesic properties [55]. Due to the complex relationship between smoking, pain, and comorbid conditions such as depression and substance use disorders, cessation counseling should be patient-centered and holistic to manage pain symptoms and nicotine withdrawal [55]. Previous studies have shown a connection between mental health and chronic pain, with each contributing to and exacerbating the other [56,57]. It has been demonstrated that chronic pain and mental health disorders are interconnected due to biological mechanisms [57], so it is surprising that we did not find a significant association between the two. Of course, encouraging smoking cessation for current smokers to improve their health should be encouraged, as should monitoring of mental health status particularly for those with extreme pain who may find it is negatively impacting their well-being.

There were several limitations (ADL, functional, and work) that were correlated to greater odds of experiencing quite a bit or extreme pain compared to those who did not report limitations. People with pain often report difficulties in work, social, recreational, and self-care activities and may struggle to maintain normal life compared to those without pain [58]. A study published in 2020 found that over half of their patients with chronic pain reported limitations in completing their ADLs, and that limitations were positively correlated with the number of pain sites and pain intensity [59]. They also found that those who reported a negative perception of the effect of pain on their ADLs were more likely to have negative social relationships [59]. There was no statistically significant association between those reported limitations of instrumental activities of daily living and experiencing quite a bit or extreme pain. Instrumental ADLs are activities that allow an individual to live independently in a community and typically require more complex thinking or organizational skills. It is possible that those who do experience limitations in instrumental ADLs require additional assistance in care homes and therefore would not have been included in the data gathered through the MEPS household query. To minimize the disabling impact that pain has on a patient’s family, work, and social life, understanding the development of limitations could help guide future prevention and treatment initiatives.

Limitations of the study include the reliance on self-reported and subjective responses for pain interference, which could be affected by recall bias. The pain interference variable was constructed based on an item querying participants about their pain interference over the past four weeks. This item is only included in two rounds of the MEPS, so responses may vary depending on the timing of the question. Future research could identify a different dataset with a more precise measure of pain to compare the findings of this study with other definitions of pain. Additionally, the cross-sectional nature of this database study does not allow for causal inference; rather, only a statistical association between the number of comorbid conditions, demographic, economic, health, and limitation variables and pain at one point in time can be established. It is not known whether pain precedes or follows multimorbidity. Furthermore, the multimorbidity variable was developed using only common conditions available in the dataset. The inclusion or exclusion of specific conditions may have influenced the results. For example, joint pain or arthritis are likely highly correlated with pain. Future research could refine the comorbidity variable by including additional comorbid conditions and assess associations between specific conditions or combinations of conditions. Future research could be conducted using different data sources to assess the relationship between other variables that may be associated with pain that were not available in MEPS, such as other socioeconomic characteristics, specific occupational hazards, environmental factors, or genetic predispositions. Additional research could also involve a longitudinal study where predictor variables are known to occur before the outcome variable (i.e., multimorbidity). Finally, subgroup analyses could be conducted to investigate any relationships that may exist between subgroups of the population, e.g., specific age groups or sex. Strengths of the study include the large number of variables accounted for in the analysis and the use of nationally representative weighting that provide good evidence of external validity to the US adult population with some degree of pain. The implications of these findings cannot be extrapolated beyond this population.

## 5. Conclusions

In conclusion, this study found that comorbidity was associated with greater odds of experiencing quite a bit or extreme pain. Several characteristics were associated with experiencing pain, including lower education, unemployment, low income, poor overall health status, and irregular physical activity. Those with limitations in activities of daily living were also more likely to experience pain, as well as those with functional or work limitations compared to those without limitations. These findings may assist practitioners in better understanding and supporting individuals experiencing pain. Future research could delve deeper into these relationships and examine whether combinations of comorbid conditions affect this relationship in different ways.

## Figures and Tables

**Table 1:** Characteristics of United States adults in the weighted study population (N = 89,314,769).

Variables		Quite a Bit/Extreme Pain N = 1446 Weighted % (95% Confidence Interval)	Little/Moderate Pain N = 4834 Weighted % (95% Confidence Interval)	*p*
Number of comorbid conditions	≥6	19.3 (16.5, 22.0)	6.4 (5.6, 7.2)	<0.0001
	5	13.3 (11.1, 15.4)	6.6 (5.8, 7.5)	
	4	17.1 (14.9, 19.3)	11.8 (10.7, 12.9)	
	3	16.9 (14.3, 19.5)	16.2 (14.9, 17.6)	
	2	17.1 (14.5, 19.8)	20.2 (18.7, 21.7)	
	1	12.5 (9.8, 15.2)	22.4 (20.8, 23.9)	
	0	3.8 (2.3, 5.3)	16.5 (14.8, 18.1)	
Demographic variables:				
Age	≥65 years	42.1 (38.6, 45.7)	33.6 (31.7, 35.5)	<0.0001
	40–64 years	44.1 (40.5, 47.7)	44.1 (42.3, 46.0)	
	18–39 years	13.8 (10.9, 16.6)	22.3 (20.5, 24.1)	
Sex	Male	40.8 (37.8, 43.9)	45.8 (44.3, 47.3)	0.0043
	Female	59.2 (56.1, 62.2)	54.2 (52.7, 55.7)	
White race	Yes	78.6 (75.5, 81.7)	79.6 (77.5, 81.8)	0.5012
	No	21.4 (18.3, 24.5)	20.4 (18.2, 22.5)	
Hispanic	Yes	10.3 (7.3, 13.2)	12.2 (10.6, 13.8)	0.1974
	No	89.8 (86.8, 92.7)	87.8 (86.2, 89.4)	
Economic variables:				
Education	≤High school	55.1 (51.2, 58.9)	40.0 (37.7, 42.2)	<0.0001
	>High school	44.9 (41.1, 48.8)	60.0 (57.8, 62.3)	
Employment	Unemployed	74.7 (71.4, 78.1)	40.1 (38.2, 42.1)	<0.0001
	Employed	25.3 (21.9, 28.6)	59.9 (57.9, 61.8)	
Health provision	Private	42.5 (38.9, 46.2)	64.3 (62.2, 66.5)	<0.0001
	Public	54.3 (50.7, 57.9)	30.1 (28.3, 31.9)	
	None	3.2 (1.6, 4.7)	5.6 (4.5, 6.7)	
Marriage	Married	44.3 (40.8, 47.8)	55.3 (53.4, 57.2)	<0.0001
	Not married	55.7 (52.2, 59.2)	44.7 (42.8, 46.6)	
Income	Low	49.1 (45.4, 52.8)	26.1 (24.2, 28.1)	<0.0001
	Mid-high	50.9 (47.2, 54.6)	73.9 (71.9, 75.8)	
Health variables:				
Good overall health	Yes	42.5 (39.2, 45.8)	82.9 (81.5, 84.3)	<0.0001
	No	57.5 (54.2, 60.9)	17.1 (15.7, 18.5)	
Good mental health	Yes	67.8 (64.4, 71.2)	86.6 (85.2, 88.1)	<0.0001
	No	32.2 (28.8, 35.6)	13.4 (11.9, 14.8)	
Regular physical activity	Yes	30.8 (27.4, 34.2)	49.1 (47.0, 51.2)	<0.0001
	No	69.2 (65.8, 72.6)	50.9 (48.8, 53.0)	
Current smoker	Yes	19.8 (16.8, 22.8)	13.1 (11.8, 14.5)	<0.0001
	No	80.2 (77.2, 83.2)	86.9 (85.5, 88.2)	
Limitation variables:				
Activities of daily living limitation	Yes	12.2 (9.8, 14.5)	1.6 (1.2, 2.0)	<0.0001
	No	87.8 (85.5, 90.2)	98.4 (98.0, 98.8)	
Instrumental activities of daily living limitation	Yes	18.6 (15.8, 21.3)	3.7 (3.1, 4.3)	<0.0001
	No	81.4 (78.7, 84.2)	96.3 (95.7, 96.9)	
Functional limitation	Yes	63.1 (59.7, 66.5)	18.9 (17.6, 20.3)	<0.0001
	No	36.9 (33.5, 40.3)	81.1 (79.7, 82.4)	
Work limitation	Yes	53.4 (49.9, 57.0)	11.3 (10.3, 12.4)	<0.0001
	No	46.6 (43.0, 50.1)	88.7 (87.6, 89.7)	

Differences between groups compared with chi-squared tests.

**Table 2:** Comorbid conditions of United States adults in the study organized from most to least common condition.

Chronic Condition	Weighted Percentage (95% Confidence Interval)
Hypertension	85.4 (83.7, 87.1)
Arthritis	46.7 (44.8, 48.5)
Hypercholesterolemia	46.1 (44.2, 47.9)
Joint pain	43.0 (40.5, 45.6)
Asthma	18.6 (17.3, 20.0)
Diabetes	18.5 (17.3, 19.7)
Other heart disease	18.0 (16.7, 19.3)
Cancer	16.3 (15.0, 17.7)
Coronary heart disease	8.8 (7.9, 9.6)
Stroke	7.1 (6.3, 7.8)
Myocardial infarction	6.8 (6.0, 7.7)
Angina	4.3 (3.6, 5.1)
Emphysema	2.8 (2.4, 3.2)
Chronic bronchitis	2.1 (1.7, 2.5)

**Table 3:** Associations between comorbid conditions and odds ratio for quite a bit or extreme pain among United States adults in the study in logistic regression analyses.

Characteristic	Unadjusted Odds Ratio (95% Confidence Interval)	Adjusted Odds Ratio (95% Confidence Interval)
≥6 vs. 0 comorbid conditions	**13.091 (8.082, 21.202)**	**3.440 (1.874, 6.314)**
5 vs. 0 comorbid conditions	**8.637 (5.316, 14.033)**	**2.827 (1.497, 5.336)**
4 vs. 0 comorbid conditions	**6.280 (4.004, 9.847)**	**2.480 (1.396, 4.404)**
3 vs. 0 comorbid conditions	**4.503 (2.875, 7.053)**	**2.392 (1.361, 4.202)**
2 vs. 0 comorbid conditions	**3.667 (2.289, 5.876)**	**2.186 (1.245, 3.837)**
1 vs. 0 comorbid conditions	**2.415 (1.480, 3.940)**	**1.916 (1.088, 3.376)**
Demographic variables:		
Age, ≥65 vs. 18–39 years		**0.526 (0.365, 0.758)**
Age, 40–64 vs. 18–39 years		0.748 (0.538, 1.040)
Sex, male vs. female		0.926 (0.781, 1.098)
White race, yes vs. no		1.186 (0.938, 1.501)
Hispanic, yes vs. no		0.901 (0.609, 1.333)
Economic variables:		
Education, ≤high school vs. >high school		**1.340 (1.091, 1.646)**
Employment, unemployed vs. employed		**1.931 (1.493, 2.500)**
Health provision, private vs. none		1.150 (0.582, 2.273)
Health provision, public vs. none		1.200 (0.633, 2.277)
Marriage, married vs. not married		1.081 (0.891, 1.310)
Income, low vs. mid-high		**1.264 (1.030, 1.551)**
Health variables:		
Good overall health, yes vs. no		**0.365 (0.302, 0.442)**
Good mental health, yes vs. no		0.807 (0.634, 1.026)
Regular physical activity, yes vs. no		**0.802 (0.655, 0.983)**
Current smoker, yes vs. no		1.040 (0.817, 1.325)
Limitation variables:		
Activities of daily living limitation, yes vs. no		**1.681 (1.121, 2.520)**
Instrumental activities of daily living limitation, yes vs. no		0.938 (0.674, 1.305)
Functional limitation, yes vs. no		**2.680 (2.169, 3.311)**
Work limitation, yes vs. no		**2.101 (1.635, 2.700)**

Statistically significant results identified in bold. Unadjusted model: Wald ≤ 0.0001. C-statistic = 0.667. Adjusted model: Wald ≤ 0.0001. C-statistic = 0.824.

## Data Availability

Data are available from the corresponding author upon reasonable request.
